# Association of *LOXL1* polymorphisms with pseudoexfoliation in the Chinese

**Published:** 2009-06-04

**Authors:** Kelvin Y.C. Lee, Su Ling Ho, Anbupalam Thalamuthu, Anandalakshmi Venkatraman, Divya Venkataraman, Don C.K. Pek, Tin Aung, Eranga N. Vithana

**Affiliations:** 1 Singapore National Eye Centre, Singapore; 2 Department of Ophthalmology, Tan Tock Seng Hospital, Singapore; 3 Genome Institute of Singapore, Singapore; 4 Singapore Eye Research Institute, Singapore; 5 Department of Ophthalmology, Yong Loo Lin School of Medicine, National University of Singapore, Singapore

## Abstract

**Purpose:**

Single nucleotide polymorphisms (SNPs) within the lysyl oxidase like-1 gene (*LOXL1*; rs1048661 and rs3825942) were found to confer risk to pseudoexfoliation glaucoma (XFG) through the pseudoexfoliation syndrome (XFS) in Nordic, Caucasian, and two Asiatic populations (Indian and Japanese). The prevalence (0.2%–0.7%) of XFS in the Chinese is considerably lower compared to Nordic populations. The aim of this study was to determine the association of *LOXL1* in Chinese subjects with XFS/XFG.

**Methods:**

Chinese subjects with clinically diagnosed XFS/XFG and normal controls were recruited. Genomic DNA was extracted, and the two *LOXL1* SNPs (rs1048661 and rs3825942) were genotyped by bidirectional sequencing. Allele and genotype frequencies were compared between cases and unrelated controls using PLINK. Linkage disequilibrium (LD) calculations and haplotype association analysis were done using the Haploview package and WHAP package, respectively.

**Results:**

Sixty-two Chinese patients (17 XFG and 45 XFS) and 171 Chinese controls were studied. The G allele of *LOXL1* SNP rs3825942 was moderately associated (OR=10.97, p=0.0018) with pseudoxfoliation in the Chinese. The frequency of the G allele of rs1048661 was not significantly different in cases compared to controls (p=0.142) in the allelic association test. However, the genotype test showed marginal association for rs1048661 (p=0.030). Only three haplotypes were observed (T-G, G-G, and G-A) with G-G as a risk haplotype (p=0.0034) and G-A as a protective haplotype (p=0.00039). T-G, which was a risk haplotype in the Japanese, was not associated with XFG in the Chinese (p=0.124).

**Conclusions:**

Polymorphisms in *LOXL1* confer risk to XFS/XFG in the Chinese. The lower incidence of XFS compared to other populations suggests additional genetic or environmental factors to have a major influence on the phenotypic expression of XFS in the Chinese. The G allele of rs3825942 has been shown to be associated with XFS/XFG in all populations studied to date.

## Introduction

Pseudoexfoliation syndrome (XFS) is the most common identifiable cause of open-angle glaucoma worldwide [1]. It is a condition characterized by abnormal accumulation of microfibrillar deposits on the surfaces of the pupillary border, anterior chamber angle, anterior lens capsule, ciliary body, and zonular fibers [2]. The prevalence of XFS increases with age [3], and worldwide prevalence rates have been found to vary in different populations [4-8]. XFS has been reported to be uncommon in Chinese people with the prevalence of 0.2% reported in Chinese Singaporean adults aged 40 years and older [9,10].

XFS is associated with ocular [11] and systemic [12-16] manifestations including a reported conversion rate of 44% to pseudoexfoliation glaucoma (XFG) over 15 years [17]. XFG has a worse prognosis than primary open-angle glaucoma (POAG) with high resistance to medical therapy and rapid progression of glaucomatous optic neuropathy [18].

A recent study demonstrated the association of XFS/XFG with three single nucleotide polymorphisms (SNPs), rs1048661 (R141L), rs3825942 (G153D), and rs2165241 (intronic), located in the first exon of the lysyl oxidase-like 1**gene (*LOXL1*) on chromosome 15q24.1 [19]. The two non-synonymous SNPs, rs1048661 and rs3825942 were highly associated with XFG (OR=2.46 and 20.10, respectively). Subsequent studies replicated the association of *LOXL1* SNPs with XFS/XFG in different populations including Caucasians, Germans, Italians, Central Europeans, Indians, and Japanese [20-30]. The association of *LOXL1* SNPs seems to be confined to XFS/XFG as studies on POAG patients including the Chinese population did not report any significant association [31-34].

Up to now, only two Asian populations, Indian and Japanese, had reported associations with *LOXL1* and XFS [26,28]. While the Indian population showed similar allelic associations with Caucasians, the Japanese, which we reported previously, had a reversal of the risk allele in rs1048661 [28]. It is unknown if other Asian populations like the Chinese have similar associations of *LOXL1* with XFS. Hence, the aim of our study was to evaluate the hitherto untested association of the *LOXL1* SNPs rs1048661 and rs3825942 in Singaporean Chinese subjects with XFS/XFG.

## Methods

### Study subjects

Chinese patients with clinically diagnosed XFS/XFG and normal Chinese controls were recruited from two tertiary eye care centers, Singapore National Eye Centre and Tan Tock Seng Hospital, in Singapore. Written informed consent was obtained from all subjects, and the study protocol had the approval of the hospitals’ ethics committees and was performed according to the tenets of the Declaration of Helsinki.

All subjects underwent detailed ophthalmic examinations by ophthalmologists that included slit-lamp biomicroscopic examination, gonioscopy, dilated examination of the lens, and funduscopy. Subjects with XFS were defined as those with clinical evidence of pseudoexfoliation at the pupil margin, anterior lens surface, or other anterior segment structures with an intraocular pressure (IOP) of less than 21 mmHg and no clinical evidence of glaucomatous optic neuropathy. Subjects with XFG were defined as those with clinical evidence of XFS and glaucomatous optic neuropathy (defined as loss of neuroretinal rim with a vertical cup:disc ratio of greater than 0.7) with compatible visual field loss. Chinese subjects with a normal anterior segment and optic nerve examination and without clinical signs of XFS/XFG were recruited as controls.

### Genotyping

Genomic DNA was extracted from peripheral white blood cells of all subjects. The genotypes of *LOXL1* SNPs rs1048661 and rs3825942 were determined by polymerase chain reaction (PCR) followed by bidirectional sequencing as described previously [28].

### Statistical analysis

Fisher’s exact tests were used to test the allelic and genotypic associations of all the SNPs with XFG and XFS. Hardy–Weinberg equilibrium (HWE) of the genotypic frequencies among cases and separately among the controls was also examined. PLINK [35] was used to do the Fisher’s exact test and the logistic regression adjusting for other covariates and SNPs. Linkage disequilibrium (LD) calculations were done using the Haploview package [36]. Haplotype association analysis was performed using the WHAP package [37]. Joint associations of all the haplotypes (global test) and haplotype specific (HS) associations were performed using this program. The haplotype specific test of association was used to examine the independent effect of any specific haplotype. For this test, none of the haplotypes was used under the null model, and the specific haplotype was entered under the alternative model. A likelihood ratio test was then constructed to assess the significance of the haplotype specific effect. Assuming the highly prevalent haplotype as the base line, each of the other haplotype was also compared with the base-line haplotype using a logistic regression model. The power of our study to detect associations was computed using the QUANTO software [38].

## Results

Sixty-two Chinese patients (45 XFS and 17 XFG) and 171 Chinese controls were recruited for the study. The mean age of patients (XFS and XFG) was 74.7±7.7 years (age range from 61 to 93), and for normal controls, the mean age was 67.4±5.6 (age range from 58 to 88). The difference in age between cases and controls was found to be significant (p=5.853x10^−10^), but the gender frequencies were not (p=0.8824). The demographic characteristics of the study subjects are shown in Table 1.

**Table 1:** Demographic features of subjects in this study.

**Features**	**Combined pseudoexfoliation XFS (45) + XFG (17) [n=62]**	**Controls (n=171)**
**Age (years)**		
mean±SD	74.7±7.7	67.42±5.6
Range	61–93	58–88
**Gender**		
Male	30 (48.39%)	80 (46.78%)
Female	32 (51.61%)	91 (53.22%)

The demographic features of the 62 Chinese pseudoexfoliation study subjects and 171 controls are shown. The age range and mean age with standard deviation (SD) are shown for each cohort. The difference in age between cases and controls was significant (p=5.853x10^−10^), but the gender frequencies were not (p=0.8824; Fisher’s exact test). Abbreviations: XFS=pseudoexfoliation syndrome; XFG=pseudoexfoliation glaucoma; n=number of subjects.

The distribution of the allele frequencies for SNPs rs1048661 and rs3825942 within *LOXL1* are shown in Table 2. The Hardy–Weinberg equilibrium was observed in the genotype frequencies for patients and controls. The SNPs rs1048661 and rs3825942 were in strong linkage disequilibrium (D’=1, 95% CI: 0.71–1.0). The G allele of SNP rs3825942 of *LOXL1* was moderately associated (OR=10.97, p=0.0018) with pseudoexfoliation in the Chinese while the frequency of the G allele of rs1048661 was not significantly different in cases compared to controls (p=0.142). The age- and sex-adjusted OR for developing pseudoexfoliation and for having the G allele at SNP rs3825942 was 16.56 (95% CI: 1.86–144.93, p=0.012). The genotype analysis showed only a marginal association with rs1048661 (p=0.0304).

**Table 2:** Distribution of *LOXL1* polymorphisms in pseudoexfoliation and control subjects.

**SNP**	**Allele**	**Allele count (frequency)**		**Allele association (p value)**	**OR** **[95% CI]**	**Genotype**	**Genotype count (frequency)**		**Genotype association (p value)**	**p value** **OR [95% CI]**
		**XFS/XFG Cases (n=62)**	**Controls (n=171)**				**XFS/XFG cases (n=62)**	**Controls (n=171)**		
rs1048661	*G	65 (0.524)	152 (0.444)	0.142	1.38 [0.91- 2.08]	G-G	20 (0.323)	29 (0.169)	0.03	0.0173** 2.33 [1.2–4.54]
	T	59 (0.476)	190 (0.556)			T-G	25 (0.403)	94 (0.550)		
						T-T	17 (0.274)	48 (0.281)		
rs3825942	A	1 (0.008)	28 (0.082)	0.0018	10.97 [1.48- 81.49]	G-G	61 (0.984)	143 (0.84)	0.0013	0.0013 11.94 [1.59–89.78]
	*G	123 (0.992)	314 (0.918)			G-A	1 (0.016)	28 (0.16)		
						A-A	0 (0.00)	0 (0.00)		

Allelic and genotype association testing results of rs1048661 and rs3825942 in *LOXL1* for Chinese cases with combined pseudoexfoliation syndrome and controls are shown. The single asterisk indicates the risk allele of each SNP. The double asterisk indicates the p values and OR values for G-G versus T-G+T-T of rs1048661. Abbreviations: SNP=single nucleotide polymorphism; XFS=pseudoexfoliation syndrome; XFG=pseudoexfoliation glaucoma; n=number of subjects; OR=odds ratio; CI=confidence interval.

The haplotype analysis of *LOXL1* polymorphisms in pseudoexfoliation and control subjects are shown in Table 3. Only three of the four possible haplotypes (T-G, G-G, and G-A) were detected in the haplotype analysis. The G-G haplotype was a risk haplotype (OR=1.92, p=0.0034) while the G-A haplotype was observed to be protective (OR=0.08, p=0.00039). The T-G haplotype was not associated with pseudoexfoliation in the Chinese (p=0.124). The two-locus haplotype frequencies in Chinese, Japanese, and Caucasian that are based on SNPs rs1048661 and rs3825942 are shown in Table 4. We have also calculated the haplotype diversity, a measure of the uniqueness of a particular haplotype in a given population, for cases and controls of the three populations [39]. Reflecting the fact that only three haplotypes are present, the haplotype diversity values were also similar within the controls of all three populations, although the frequency distribution of haplotypes was somewhat different. The predominance of the T-G haplotype in the Japanese affected with pseudoexfoliation was reflected in the low haplotype diversity value (H=0.102) for this sample. The allele frequencies of the two non-synonymous SNPs of *LOXL1* in different populations are shown in Table 5.

**Table 3:** Haplotype analysis of *LOXL1* polymorphisms in pseudoexfoliation and control subjects.

rs1048661	rs3825942	**Haplotype frequency**		HS test* p value	OR* [95%CI]	p value**	OR** [95%CI]
		**XFS/XFG total**	**Controls**				
T	G	0.476	0.556	0.124	1.39 [0.91–2.12]	-	-
G	G	0.516	0.363	0.0034	1.92 [1.25–2.96]	0.018	1.7 [1.1–2.63]
G	A	0.008	0.082	0.00039	0.08 [0.01–0.62]	0.031	0.11 [0.01–0.80]

All haplotypes with frequency greater than 1% in the combined case and control sample are shown in the table. The XFS/XFG total indicates pseudoexfoliation with and without glaucoma. The single asterisk indicates haplotype specific (HS) testing. This test is a comparison of a specific haplotype with the other two. The double asterisk indicates the p values and OR values derived from comparing each haplotype with the base-line haplotype (T-G). p values for HS testing are based on 10,000 permutations. Abbreviations: XFS=pseudoexfoliation syndrome; OR=odds ratio; CI=confidence interval; XFG=pseudoexfoliation glaucoma.

**Table 4:** Frequencies of the two-locus haplotype among three different populations.

rs1048661	rs3825942	**Haplotype frequency**					
		**Chinese**		**Japanese [**28**]**		**Caucasian [**21**]**	
		**XFS/XFG (n=62)**	**Controls (n=171)**	**XFS/XFG (n=209)**	**Controls (n=172)**	**XFS/XFG (n=86)**	Controls (n=2087)
T	G	0.476	0.556	0.947	0.506	0.22	0.34
G	G	0.516	0.363	0.039	0.365	0.74	0.51
G	A	0.008	0.082	0.014	0.129	0.05	0.15
Haplotype diversity (±SE)		0.511 (± 0.01)	0.554 (± 0.014)	0.102 (±0.02)	0.596 (± 0.014)	0.404 (±0.037)	0.602 (±0.004)

The two-locus haplotype frequencies in the Chinese, Japanese, and Caucasian are shown. Only one example is shown for Caucasians from a Caucasian Australian population for comparison with the two Asiatic populations. The distribution of the T-G, G-G, and G-A haplotypes were significantly different between cases and controls in both the Japanese and Caucasian studies shown here [21,28]. In our present Chinese study, only the G-G and G-A haplotypes were significantly different between cases and controls. Haplotype diversity is also given for each sample. Haplotype diversity is a measure of the uniqueness of a particular haplotype in a given population. The haplotype diversity and its variance were calculated using the formula given within the indicated reference [39].

**Table 5:** Reported allele frequencies of *LOXL1* polymorphisms in pseudoexfoliation and control subjects among different populations.

**Population**	**Phenotype**	**Allele frequency**		**Sample size**	**References**
		rs1048661 ** (G/T)**	rs3825942 ** (G/A)**		
		**G**	**G**		
Iceland	XFG	0.827	0.987	75	[19]
	Controls	0.651	0.847	14474	
Sweden	XFG	0.834	0.995	199	[19]
	Controls	0.682	0.879	198	
USA Caucasian	XFG	0.787	0.939	50	[20]
	Controls	0.665	0.844	235	
Australia	XFG, XFS	0.78	0.95	86	[21]
	Controls	0.66	0.84	2087	
Europe (German+Italian)	XFG, XFS	0.82	0.965	726	[24]
	Controls	0.652	0.851	418	
Southern India	XFG, XFS	0.72	0.92	52	[26]
	Controls	0.63	0.74	97	
Japan	XFG, XFS	0.053 (T=0.947)	0.99	209	[28]
	Controls	0.497	0.86	172	
Japan	XFG	0.005 (T=0.995)	0.995	95	[30]
	Controls	0.47	0.85	190	
Singapore (Chinese)	XFG, XFS	0.524	0.992	62	Present study
	Controls	0.444	0.918	171	
Hong Kong (Chinese)	Controls	0.472	0.876	250	[34]
African American	Controls	NA	0.599	97	[31]

Allele frequencies for the G alleles of rs1048661 and rs3825942 in pseudoexfoliation cases (with and without glaucoma) and controls among different populations are shown. The allele frequencies are shown for normal Chinese controls from Hong Kong for comparison with our present study. NA: not available.

## Discussion

Our study showed that the G allele of rs3825942 within *LOXL1* confers a 10 fold risk to XFS/XFG in the Chinese population. Genotype analysis revealed that both non-synonymous SNPs, rs3825942 and rs1048661, were associated with XFS/XFG, although the latter only marginally. This result for rs1048661 can be attributed to the limited power of our study due to small sample size. The estimated power of our study ranged between 30%–50%, calculated based on the observed range of allele frequencies, the odds ratios in our data, prevalence of 0.2%, and a 1:3 case-control ratio. Since Thorleifsson et al. [19] reported the results of a genome-wide association study of XFG, which identified three strongly associated variants of *LOXL1*, other studies have replicated the association of the two non-synonymous variants, R141L (rs1048661) and G153D (rs3825942) in different populations. These *LOXL1* replication studies in various populations have suggested rs3825942 to be the XFS-associated SNP with the G allele being the universally disease risk-associated allele. The association of rs1048661 with XFS/XFG is controversial due to several reasons. In all the Japanese studies conducted to date, the disease associated allele of rs1048661 was T instead of G as seen in Caucasian or the Scandinavian studies [27-30]. In other studies such as the one on an Asian population of Southern Indians, the sample sizes were too small to detect statistically significant associations with rs1048661 [26]. However the G allele of rs3825942 has been shown to be associated with XFS/XFG in all population studied to date.

To compare our Chinese haplotype data with haplotype data from a Japanese population and a Caucasian population, we have shown the frequencies of the two-locus haplotype for the three different populations (Table 4). Our study data conform more with what has been observed in the Caucasian and the Scandinavian studies than with other Japanese studies, even though Japanese and Chinese populations may have a more recent common population history compared to other populations [40,41]. The T-G haplotype, which was the risk haplotype in the Japanese population, was not associated with XFS in the Chinese population in our study. Moreover, the G-G haplotype, which is a risk haplotype in the Chinese as it is in Caucasian and the Scandinavian cohorts, is a protective haplotype in the Japanese. However, the frequencies of the three main haplotypes (T-G, G-G, and G-A) between Chinese and Japanese controls are very similar. The T-G haplotype with a frequency at around 50%–55% is the major haplotype in both populations whereas in the Nordic and Caucasian populations, the G-G haplotype is the dominant haplotype [18-23,28]. This discrepancy observed in the risk haplotypes between the Chinese and Japanese may partly be caused by some combination of the effects of other unlinked modifier genes and environmental effects influencing the disease penetrance in these two populations. The haplotype formed by protective alleles (T-A) was also not observed in the Chinese case-control groups, similar to data from other Asian populations (Indian and Japanese) as well as Scandinavian and Caucasian populations [19-30].

We also compared the allele frequencies for the G alleles of rs1048661 and rs3825942 in pseudoexfoliation cases (with and without glaucoma) and controls among different populations (Table 5). The minor allele frequency of rs3825942 was highest in African Americans. This may reflect geographically varying selective pressures and/or a much older population. The minor allele frequency of rs3825942 was more stable and considerably lower at approximately 15% in all non-African populations. In comparison, the minor allele frequency of rs1048661 was higher and more varied across populations. This suggests that the G153D variation may produce more deleterious alteration to the gene product than R141L and that there is higher selection against maintaining this polymorphism in populations than R141L. The high variability of the minor allele frequency of rs1048661 may also indicate the influence of factors such as genetic drift on this allele frequency. It is interesting to note that these minor alleles are protective against pseudoexfoliation in all populations studied except in the Japanese where the T allele of rs1048661 was associated with disease. The frequency of the G allele for SNP rs3825942 at 0.918 is slightly high in our control cohort. However, another study that investigated the *LOXL1* SNPs in Chinese POAG patients from Hong Kong and Beijing with a larger control cohort found the G allele frequency to be 0.876 in their control group, similar to other Caucasian populations. The higher frequency of the G allele in our Chinese cohort may be related to the small sample size. The control group allele frequencies of the non-synonymous SNPs reported by Gong et al. in another Chinese sample were generally similar to our study and may represent the migration of Southern Chinese to Singapore [34].

The prevalence of XFS/XFG varies widely across populations, ranging from 20%–25% in the Scandinavian countries of Iceland and Finland [42,43] to less than 1% in the Chinese and Japanese [7,9,10]. Interestingly, the lowest G allele frequency for rs3825942 at 0.599 has been observed in the African American population where the XFS/XFG prevalence is low at approximately 0.4% [6,31,44]. However, dissimilarities in risk allele frequencies across populations alone cannot explain the widely different prevalent rates that are observed. Similar allelic architecture in different populations with widely differing disease prevalence has also been observed [21]. This suggests other yet unidentified genetic and/or environmental factors independent of *LOXL1* that influence the penetrance of the disease.

In summary, we found an association of *LOXL1* variants with XFS/XFG in the Chinese. Our study of Chinese subjects with XFS further supports previous reports that the G allele of rs3825942 is the universal risk allele associated with XFS. While we were able to replicate similar results in the Chinese population, other genetic and/or strong environmental factors could be modulating the phenotypic expression of XFS in the Chinese, which results in a lower prevalence of the disease in this population.
